# Valorization of novel bifunctional waterborne coatings with UV irradiation resistance and antimicrobial activity

**DOI:** 10.1038/s41598-025-17291-9

**Published:** 2025-09-01

**Authors:** Walaa M. Abd El-Gawad, Hossam M. El-Masry, T. A. Zidan

**Affiliations:** 1 Department of Polymers and Pigments, National Research Centre, 33 El Buhouth St, Dokki, Giza, 12622 Egypt; 2 Chemistry of Natural and Microbial Deparment, National Research Centre, 33 El Buhouth St, Dokki, Giza, 12622 Egypt

**Keywords:** Acrylic resin, Bifunctional coatings, UV irradiation resistance, Antimicrobial activity, Environmental sciences, Chemistry, Materials science

## Abstract

This research aimed to create bifunctional acrylic waterborne coatings capable of absorbing UV radiation and resisting microbial growth. The compound 4-[2(3-acetylphenyl) diazenyl]-3,5-dimethylphenol (ADD) was incorporated into the waterborne acrylic resin at concentrations of 0.1%, 0.25%, and 0.5%. The coatings underwent characterization through scanning electron microscopy (SEM), mechanical property testing, and the CIELab color method after 500 h of UV exposure to assess their UV shielding effectiveness. Furthermore, the antimicrobial properties of both ADD powder and the coatings were evaluated against Gram-negative bacteria (*Helicobacter pylori*), Gram-positive bacteria (*Staphylococcus aureus*), and pathogenic fungi (*Candida albicans*) using the disc diffusion method. Results indicated that the coatings with 0.25% and 0.5% ADD retained their integrity, showing no cracks or color and texture changes after UV exposure. In contrast, the 0.1% ADD coating exhibited significant alterations in the a* value, revealing its susceptibility to UV damage and limited UV absorption. Positive a* values confirmed the red tint of the films. Antimicrobial activity was notable, with inhibition zones measuring 14 to 26 mm against *Staphylococcus aureus*, 11 to 21 mm against *Helicobacter pylori*, and 12 to 20 mm against *Candida albicans*. Overall, this study demonstrated that the developed coatings with ADD significantly enhance UV absorption and exhibit promising antimicrobial properties, effectively overcoming the limitations of existing commercial coatings and offering a viable solution for protecting surfaces from UV radiation and microbial contamination.

## Introduction

Recently, UV irradiation has been considered as one of the most severe causes triggering the destruction of organic coatings^1^, as this energy-rich radiation promotes the chemical change in the coatings. The radiation captured by such compounds causes photooxidative damage, which degrades the optical and physico-mechanical properties of the different materials. The chemical instability of coatings is influenced by the excited state characteristics of the materials. Strong UV light produces extremely reactive free radicals, also known as reactive oxygen species (ROS), which have the potential to degrade the coating layers, such as the hydroxyl (OH^**•**^), alkoxy (RO^**•**^), organic peroxyl (ROO^**•**^), hydroperoxyl radicals (HOO^**•**^), and superoxide radical anion (O_2_^**•−−**^)^2^. The coating layers may deteriorate as a result of the electron-transfer processes that start a chain reaction. This is why enhancing the protection performance of coatings against UV radiation is very important. The coating should not only absorb a significant portion of the UV irradiation, but it should also be UV resistant, and its physico-mechanical or optical properties should not be affected by UV^3,4^.

Acrylic resins are a mainstay in the coatings industry due to their exceptional qualities, which include their flexibility, durability, and environmental friendliness^5,6^. Because these resins are composed of acrylic monomers that have been polymerized in an aqueous solution, they are exceptionally resistant to weathering and chemical damage. However, the polymer itself is not highly effective in capturing UV irradiation; thereby, it is unable to completely shield against UV irradiation. This limitation may be eliminated through the addition of an appropriate UV absorber or shielding pigments (e.g., benzophenone derivatives)^7,8^. Pigments are important constituents in producing a colorful world. Their role is to make coloration and coverage. Organic pigments are widely utilized in several applications as textiles, coatings, and inks due to their wide color spectrum, great brightness, and powerful tinting power^8–10^.

On the other hand, the existence of numerous microorganisms in nature (e.g., viruses and bacteria) may have a negative impact on human life. The growth and accumulation of various microorganisms on the surfaces have traditionally been seen as increasing dangers by compromising health. The direct contact of humans with these microbes can result in severe infections and other ailments. Antimicrobial coatings are the major way of reducing the bacterial colonization^11^. Today, more regulations are being imposed on the advancement of bio-repellent antimicrobial coatings. For a long time, antimicrobial coatings have relied mostly on CuO, which is quite effective, but its utilization is currently restricted due to the environmental rules. Nowadays, the world is striving for environmentally friendly solutions; therefore, numerous safe alternatives with low cost, high safety, and effectiveness were proposed in the current effort to replace the previous hazardous ones^12–15^.

**Fig. 1:**
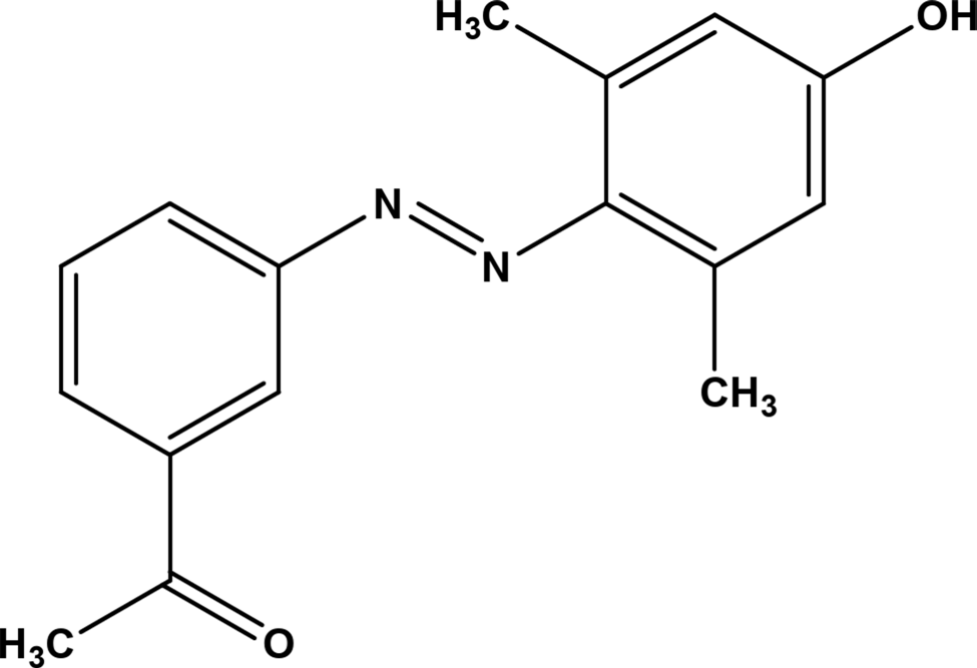
ADD structure.

Zidan et al. prepared and characterized an organic pigment, namely, 4-[2(3-acetylphenyl) diazenyl]-3,5-dimethylphenol (ADD) with the chemical structure shown in Fig. 1^16^. ADD’s structural, linear, and non-linear optical characteristics were examined with the heterojunction photodiode properties of Ag/ADD/p-Si/Al. ADD thin films showed good optical characteristics, making them suitable for usage as UV absorbers.

Therefore, the key goal of this work is to integrate ADD into acrylic resin with different ratios to prepare bifunctional eco-friendly coatings that can provide UV irradiation shielding and antimicrobial activity in a single coating layer.

## Experimental

### Materials

3-Aminoacetophenone, sodium nitrite (NaNO₂), and 3,5-dihydroxyphenol are purchased from Sigma-Aldrich, Egypt. Perimal E 822 K emulsion, which is obtained from Pachin Co. in Egypt, is a pure acrylic binder, suitable for the production of matt, sheen, or semi-gloss interior and exterior coatings. Nutrient agar medium (NA), which consists of (g/L) yeast extract 2.0, peptone 5.0, meat extract 1.0, NaCl 5.0, agar 15.0, and pH 7.4 ± 0.2, while the nutrient broth medium (NB) consists of (g/L) yeast extract 2.0, peptone 5.0, meat extract 1.0, NaCl 5.0, and pH 7.4 ± 0.2. One set of samples has the following codes [(Sample-1), (Sample-2), (Sample-3), & (Sample-Powder)]; 100.0 mL sterile conical flasks, and the following tested pathogenic microbial strains were Gram-negative bacteria [*Helicobacter pylori* (ATCC 43526)], Gram-positive bacteria [*Staphylococcus aureus* (ATCC 6538)], and finally pathogenic fungi *Candida albicans* (ATCC 10231).

### Synthesis of 4-[2(3-acetylphenyl) diazenyl]-3,5-dimethylphenol (ADD)

4-[2(3-Acetylphenyl) diazenyl] -3,5-dimethylphenol (ADD) was synthesized according to a previous work^16^. Three steps were involved in the synthesis of ADD. The first step was the reaction between 0.1 M hydrochloric acid (0.36 gm, 0.24 ml) and 3-aminoacetophenone (0.01 mol, 1.35 gm) for 5 min at 0–5 °C controlled by an ice bath. The pH of the reaction was 2. The second step was the addition of an aqueous solution of NaNO₂ (0.01 mol, 0.69 gm) to the former mixture and the reaction was maintained at 0–5 °C for additional 5 min at the same pH. The third step was the addition of 3,5-dihydroxyphenol (0.01 mol, 1.22 gm) in 0.1 M NaOH (0.4 gm in100 ml H_2_O) solution to the later mixture at the same reaction temperature. The pH of the reaction was 8. The reaction was persisted for additional one hour. After isolation of the precipitate, it was washed many times with distilled water, filtered, dryed and recrystallized from ethanol to obtain an orange-red product (ADD) with 78% yield.

### Coatings preparation

As shown in Table 1, three formulations were prepared as the organic pigment was milled with acrylic emulsion according to the proportion of pigment to binder mass fractions of 0.1%, 0.2%, and 0.5%. First, the weighted organic pigment was mixed with a definite volume of water using ultrasonic for 0.5 h to enhance the dispersion of the ADD in the formulations. After that, the acrylic resin was added to every dispersed solution and mixed using a ball mill for 2 h to prepare the coatings. Then, the coatings were filtered to make sure there were no coagulated particles. Finally, the formulations were painted on a plastic substrate using a film applicator with a thickness of 120 μm to determine their antimicrobial activity and UV resistance.

**Table 1:** The coatings formulation.

Coating	Ingredients (Wt.%)		
	0.1%	0.25%	0.5%
Water (ml)	20	20	20
Perimal E 822 K emulsion (gm)	79.9	79.75	79.5
ADD (gm)	0.1	0.25	0.5

### Evaluation of the prepared coatings

#### UV resistance

ASTM D4587-91 was used to investigate the resistance of UV irradiation, where the coats were exposed to a 4-watt UV lamp with a 245/312 nm wavelength in a black chamber at ambient temperature for 500 h. The UV irradiation resistance of the coats was evaluated before and after the exposure via CIELab (color), SEM, and FTIR techniques. Scanning electron microscopy (SEM)/energy-dispersive X-ray analysis (EDX) techniques using micro-analyzer electron probes (JEOL JX 2840) in Japan were used to determine the texture and appearance of the coat’s surface before and after the exposure to UV irradiation. Besides, Fourier transforms infrared spectroscopy (FTIR) spectra of the coats were obtained with a JASCO FTIR-4100 E FT-IR spectrometer (Japan) operating in absorption mode in the wavenumber range of 4,000–400 cm⁻¹.

#### Color measurements

The color changes of coatings because of ultraviolet irradiation were investigated using a Lovibond Tintometer RT 100 Color by the CIELab method. In this investigation, L* represents the lightness axis (0 for black, 100 for white). a* is the green (-) to red (+) axis, while b* is the blue (-) to yellow (+) axis. Moreover, the total variations (∆E) were measured according to the following equation:

$$\:(\varDelta\:\text{E})=\sqrt{{\left(\text{L}\text{*}\right)}^{2}}+\sqrt{{\left(\text{a}\text{*}\right)}^{2}}+\sqrt{{\left(\text{b}\text{*}\right)}^{2}}$$

#### Mechanical characteristics

Several ASTM standards, including hardness (ASTM D 6577) and ductility (ASTM D 5638), and impact resistance (ASTM D 2794), are used to demonstrate the elasticity and strength of the coated films before and after exposure to UV irradiation.

#### Antimicrobial measurements

The antimicrobial assessments of the produced coatings are carried out against three pathogenic strains: Gram-negative bacteria, *Helicobacter pylori* (ATCC 43526), and Gram-positive bacteria, *Staphylococcus aureus* (ATCC 6538), as well as a pathogenic fungal strain such as *Candida albicans* (ATCC 10231). All the previously pathogenic strains were prepared from fresh nutrient broth cultures incubated overnight at 37 °C. A 20 mL of sterile nutrient agar medium (NA) was inoculated separately with 25 µL of 0.5 McFarland standard (1.5 × 10^8^ CFU /ml) from the previous strains and poured on sterile petri dishes, then by using the disc diffusion method, the discs of the tested samples, were placed on the surface of those inoculated plates. These inoculated plates were placed in the refrigerator for one hour for more diffusion of the active ingredients of these discs, followed by incubation at 37 °C for 24 h, and inhibition zones of (IZ) were measured in mm^11^.

The ADD was passed to another test for determination of the minimum inhibitory concentration value (MIC) using a nutrient broth medium applying the microdilution broth method^17^. All data are presented as mean ± standard deviation. Statistical evaluation was conducted using ANOVA. Results with P-values less than 0.05 (*), 0.01 (**), 0.001 (***), and 0.0001 (****) were considered statistically significant. Each experiment was performed three times.

## Results and discussion

### Color investigation

**Fig. 2:**
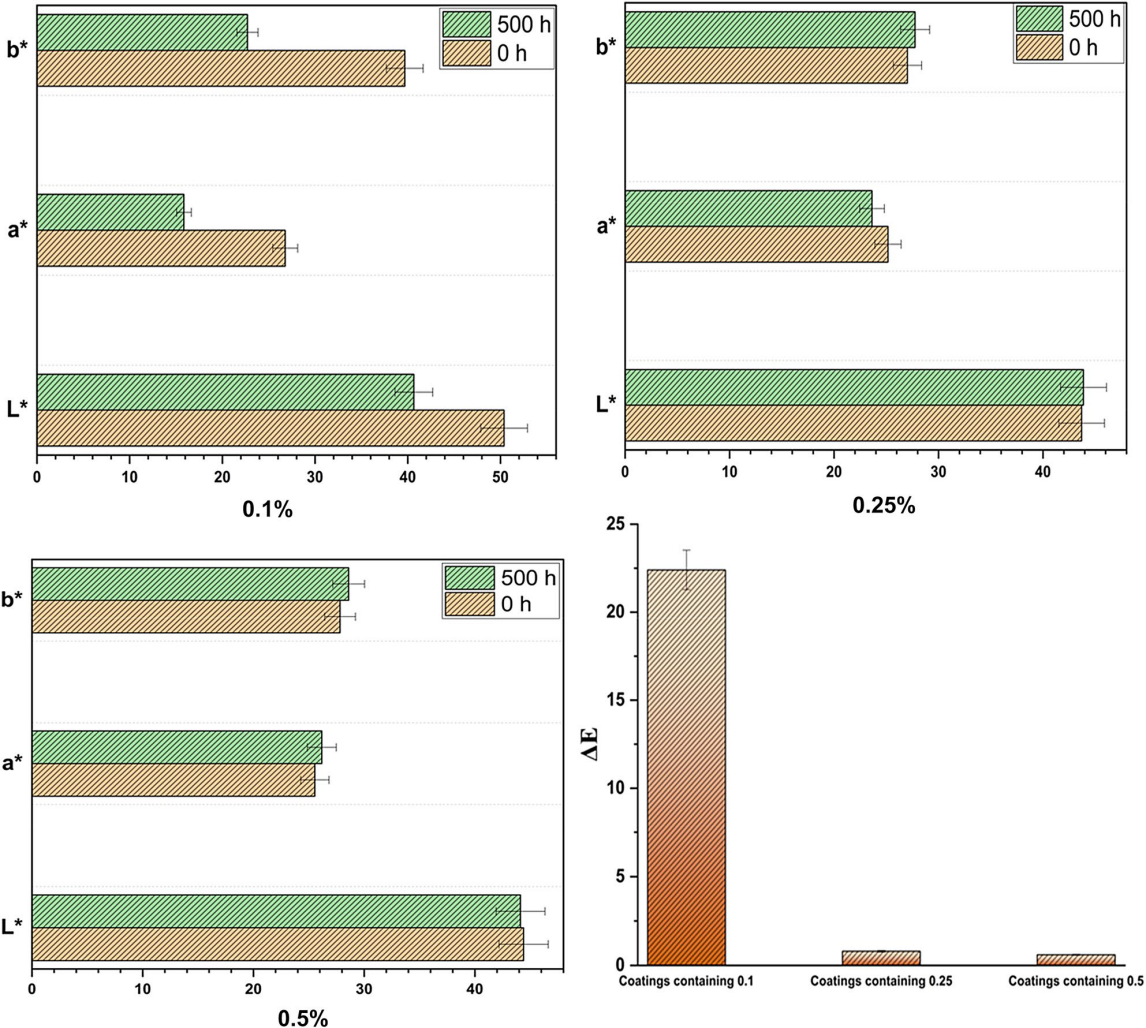
Color measurements before (0 h) and after (500 h) exposure to UV irradiation.

**Fig. 3:**
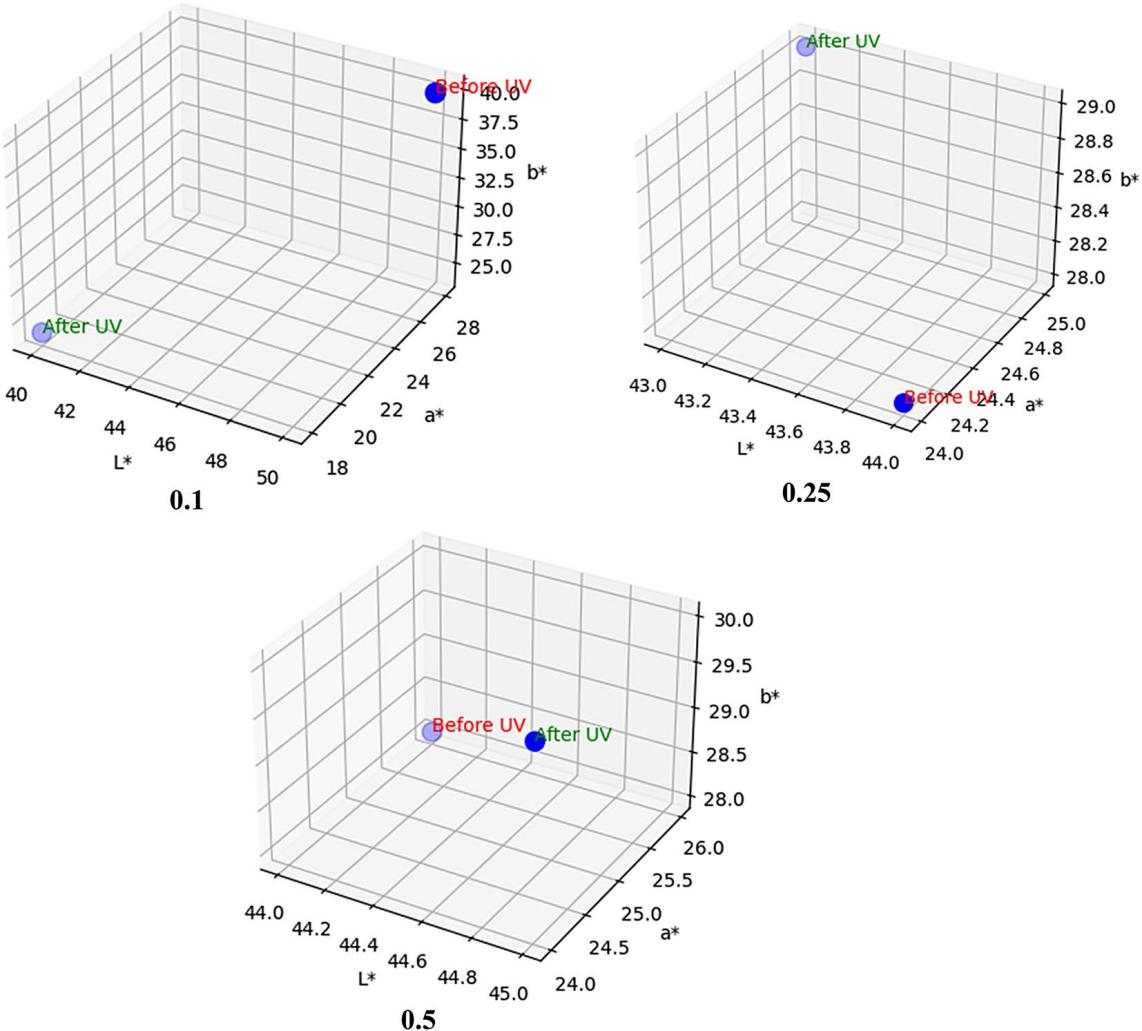
The CIELab color space before (0 h) and after (500 h) exposure to UV irradiation.

The protective effect of the prepared coatings is investigated by determining the color change after the exposure to UV irradiation during 500 h. As shown in Fig. 2, all the coatings have positive a* values, which confirms the red color of the coatings, and the a* value is increased with increasing the concentration of the organic coating. Additionally, the figure demonstrates that color change is illustrated by the a* value difference (on the green-red coordinate axis) in the CIELab system. The coating containing 0.1% shows the highest change in the a* value, which means that this ratio can’t absorb UV irradiation and is affected by UV irradiation easily. While coatings containing 0.25% and 0.5% of the organic pigment display insignificant changes in the a* values. Figure 3 shows that the distance between the two points can illustrate the degree of color change due to UV exposure. The distance between the two points in the coating containing 0.1% is long, which declares that the change is high. However, the distance between the two points in the coating containing 0.5% is short, which confirms that the change in the color is very low. These investigations provide that increasing the concentration of the organic pigment enhances the protective performance of the coatings against UV irradiation.

Usually, the color changes are attributed to the interaction between the polymeric chain and the photons produced from ultraviolet irradiation leading to photo-oxidative reactions. In Fig. 2, the color variation (∆E) of the coating with 0.1% is around 22.5%, while (∆E) values are decreased significantly with increasing the proportions of ADD^18^. The results confirm the integration of ADD into acrylic polymer with high ratios enhances the UV irradiation resistance, which may be attributed to the molecular structure of ADD. There is delocalization of electrons through the conjugated azo group (-N = N-) and the alternating double and single bonds. Accordingly, the molecule can thus absorb UV light and release the energy without decomposing. Furthermore, the molecule is further stabilized and has improved resistance to UV degradation due to the presence of aromatic rings and other functional groups. This makes ADD extremely resilient to UV light and does not fade^19,20^.

### Investigation of the change before and after UV irradiation exposure using SEM

**Fig. 4:**
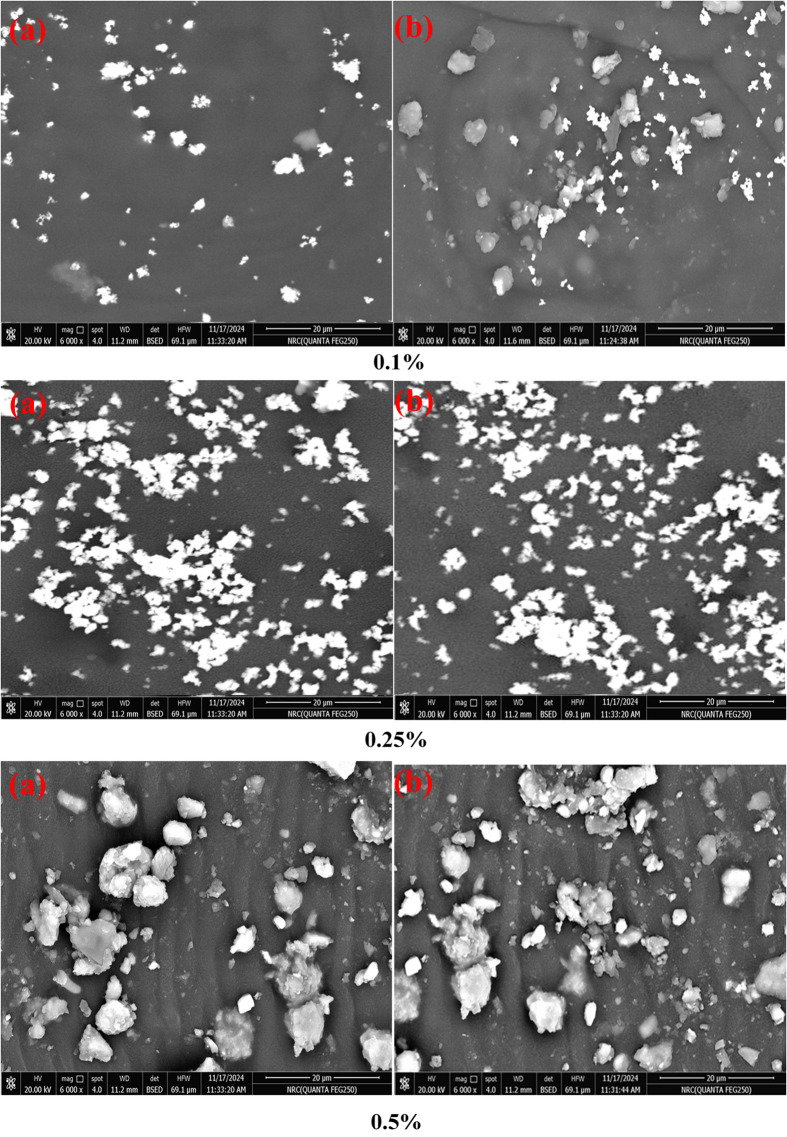
SEM images before (0 h) and after (500 h) exposure to UV irradiation.

The morphology of the coatings containing ADD organic pigments is investigated using the SEM technique before and after UV irradiation exposure during 500 h. This technique could be employed to evaluate potential surface defects. Moreover, this approach enables the identification of considerable aggregations of ADD. Figure 4 illustrates the SEM micrographs of the coatings containing ADD before and after exposure to UV irradiation. The figure shows that the film containing 0.1% of ADD has cracks that can be readily observed after the exposure, and the surface is partially covered with ADD. This may be due to the low ratio of ADD between the polymeric chains, which decreases the ability of the coating film to absorb UV irradiation; thus, ultraviolet degradation leads to diminished polymeric chain mobility, increases the brittleness of the coating, and induces micro-cracks^21,22^. In the case of coatings with 0.25% and 0.5% ADD, no changes were observed between the coated surfaces before and after UV irradiation exposure. These findings confirm the results obtained from color evaluation and declare that the UV absorption effect is increased by increasing ADD concentration. Moreover, ADD is well distributed, and no perceptible aggregates are observed in the film containing 0.25%, while ADD is agglomerated in the film containing 0.5%. However, this agglomeration doesn’t affect the UV absorption.

### FTIR analysis

**Fig. 5:**
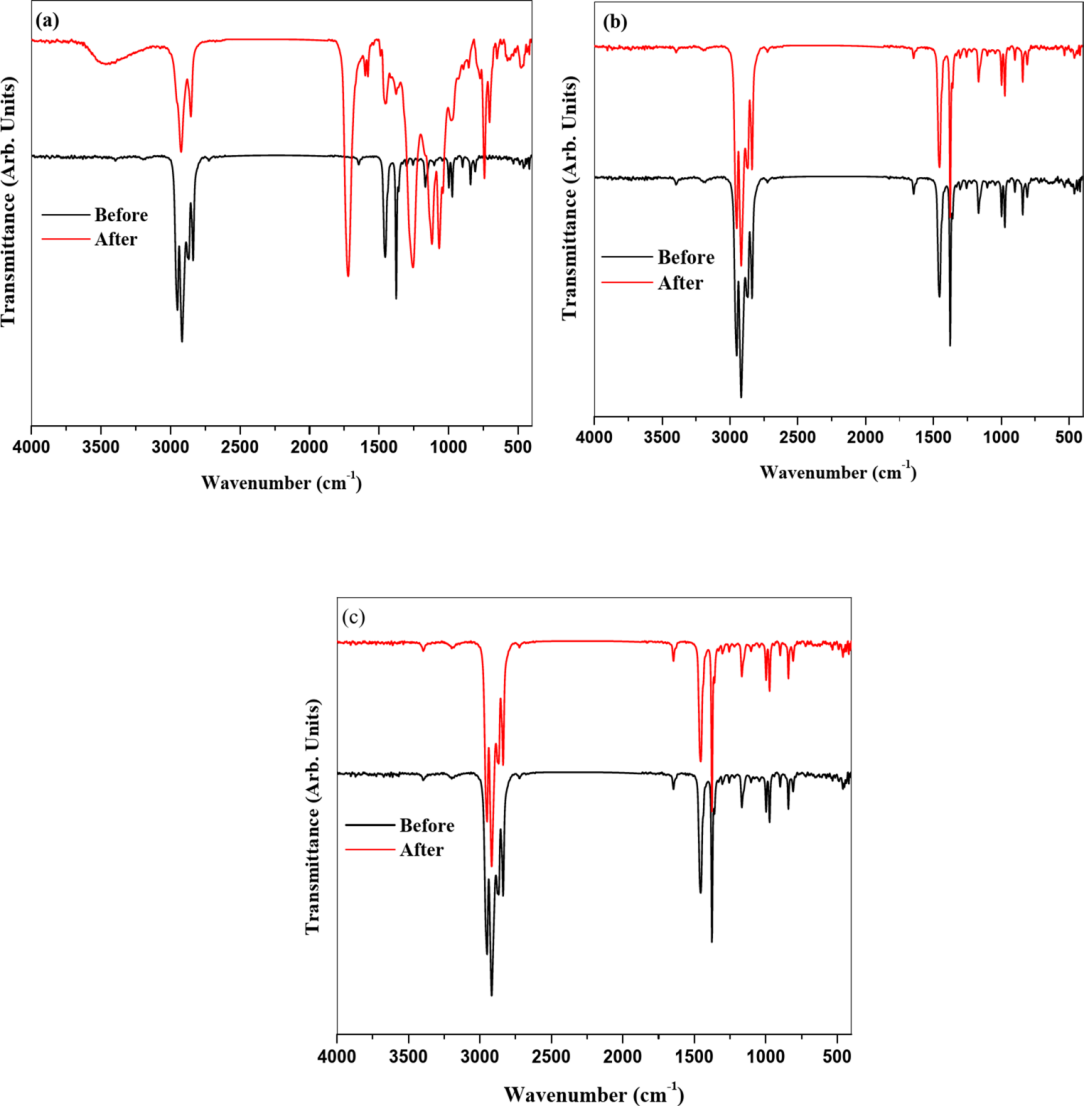
FTIR of coatings with (**a**) 0.1%, (**b**) 0.25%, and (**c**) 0.5% before and after exposure to UV irradiation.

**Fig. 6:**
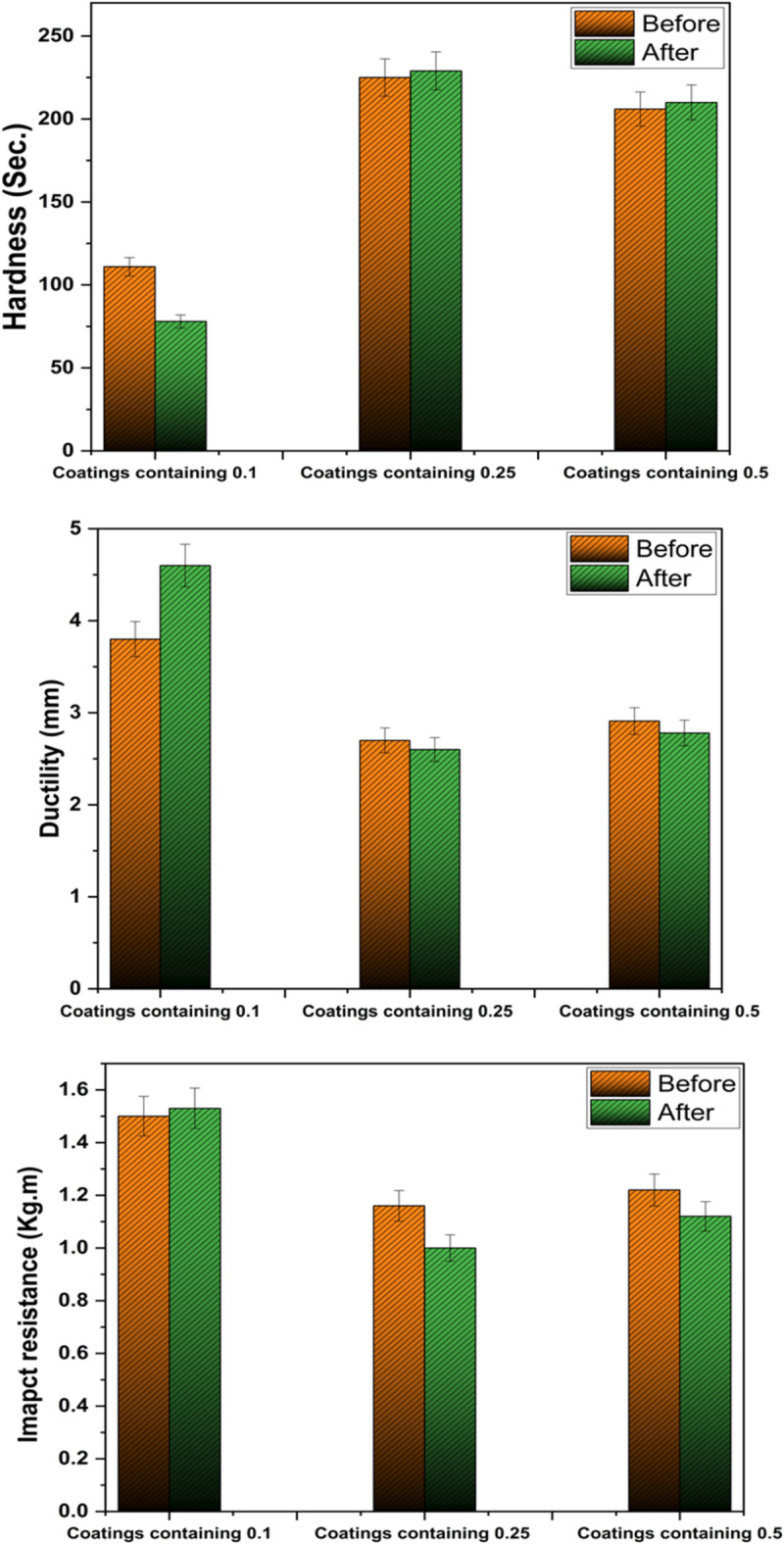
The mechanical properties before (0 h) and after 500 h of exposure to UV irradiation.

The FTIR analysis of coatings with 0.1%, 0.25%, and 0.5% ADD is investigated before and after the exposure to UV irradiation after 500 h as illustrated in Fig. 5. The FTIR for all the compositions containing both the acrylate coating and ADD before exposure to UV irradiation reveals the presence of peaks corresponding to both coating and ADD. It shows the presence of a band at 3395 cm^− 1^ corresponding to the stretching vibrations of the hydroxyl group of ADD. A vibrational band at 3198 cm^− 1^ is observed for the stretching vibrations of aromatic –CH group of ADD and = CH of acrylate. The stretching vibrations of the aliphatic –CH3 group of both ADD and acrylate are observed at 2947 cm^− 1^, 2914 cm^− 1^, 2871 cm^− 1^, and 2840 cm^− 1^, respectively. The carbonyl group stretching vibrations of ADD are observed at 1642 cm^− 1^. The phenyl group vibrations present at 1452 cm^− 1^^16^. For acrylate coating, the 1140 cm⁻¹ may be attributed to C–O–C stretching vibrations. C–O stretching modes appeared at 1237 cm⁻¹^23^. The disappearance of the peak represents the carbonyl group of acrylate ester confirms the good chemical interaction between the acrylate coating and ADD. After exposure to UV irradiation, the results demonstrate that only coating with 0.1% showed a change in FTIR bands and a new peak appeared at 1724 cm⁻¹. This confirms breaking of the chemical interaction between the coating and ADD by exposure to UV irradiation, so the carbonyl ester of the acrylate coating appeared at 1724 cm⁻¹. While there are no changes noticed in coatings with both 0.25% and 0.5% ratios and this indicates that the chemical interaction between the coating and ADD doesnot be affected by exposure to UV irradiation. This data approves the results obtained from the SEM and color investigations (Fig. 6).

### Mechanical properties

**Fig. 7:**
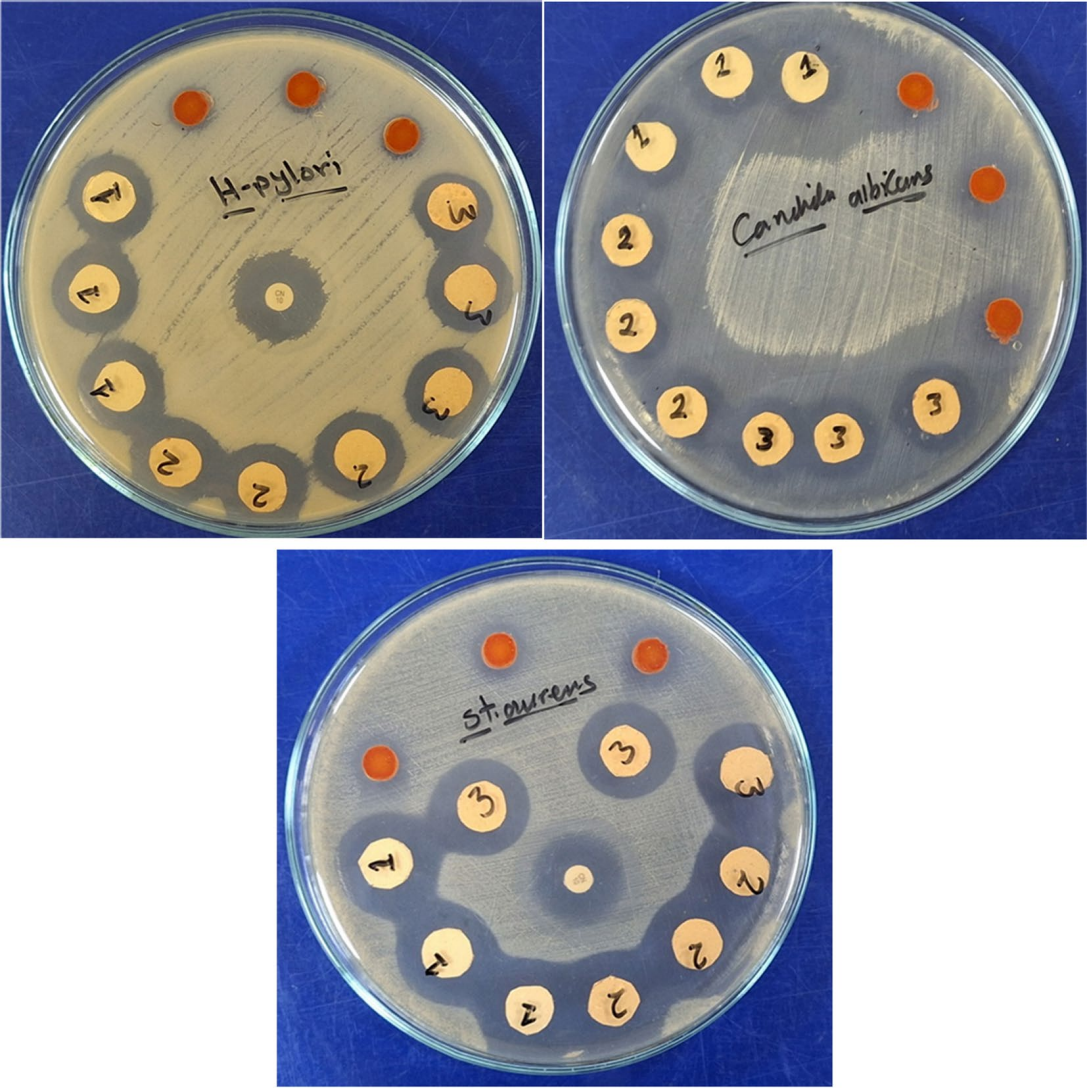
The antimicrobial activity of the coatings.

Figure 7 indicates the mechanical properties of the coatings containing ADD. The findings demonstrate that the hardness of coatings with 0.25% and 0.5% of ADD is the best. As the hardness values of coatings with 0.25% and 0.5% are almost 225 and 217 s., respectively, while this of film has 0.1% is 115 s. The good hardness of film containing 0.25% of ADD may be related to the well distribution of the ADD between the polymeric chains. This leads to the formation of tightly packed films free of holes; therefore, they have a lower susceptibility to fracturing and possess enhanced hardness. Moreover, because of the good hardness of the films with 0.25% and 0.5%, their ductility and impact resistance are lower than that of the film with 0.1%^24^.

Regarding the change in the mechanical properties before and after exposure to UV irradiation for 500 h, the results show that the hardness, ductility, and impact resistance of the film with 0.1% of ADD are considerably changed after the exposure. While no significant change was observed in the case of coatings with 0.25% and 0.5% of ADD.

### Antimicrobial activity

**Fig. 8:**
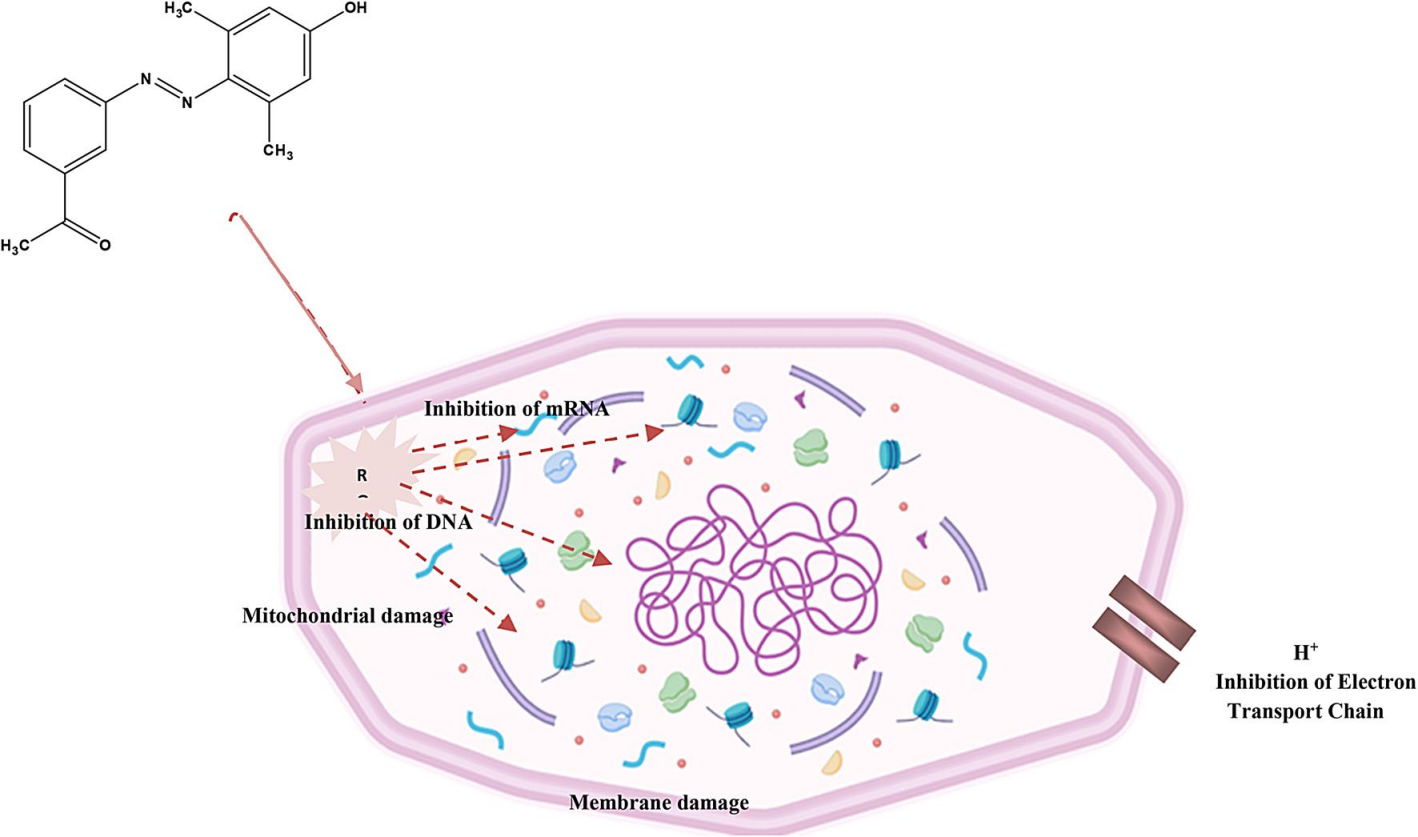
Schematic diagram of ADD antimicrobial activity.

**Fig. 9:**
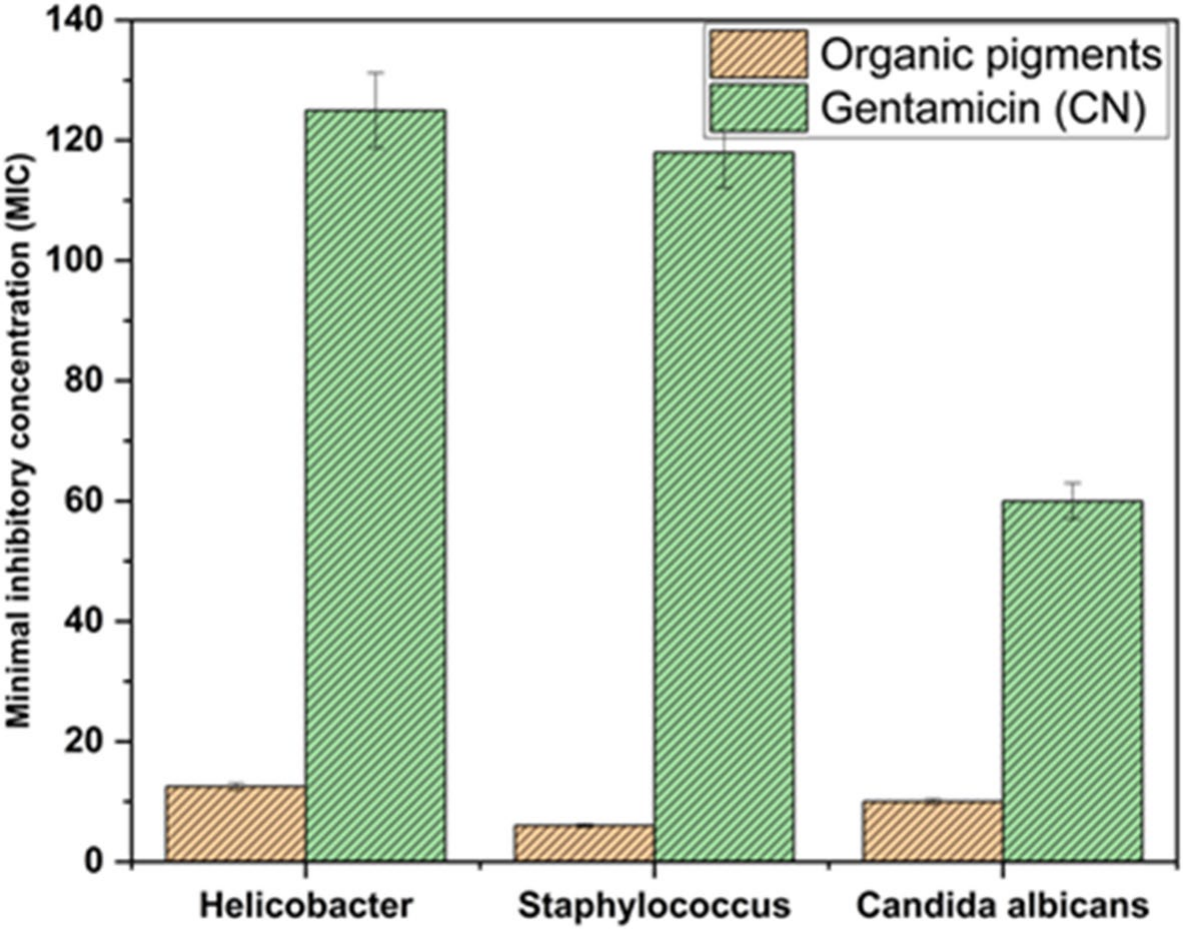
MIC of ADD.

Herein, the antimicrobial activity of ADD organic pigment and coatings with ADD was evaluated against *Staphylococcus*, *Helicobacter pylori*, and *Candida albicans* (pathogenic fungi). It is shown in Fig. 8; Table 2 that ADD and coatings have excellent antimicrobial activity by performing the disc diffusion approach. The inhibition zone diameter ranged from 14 to 26 mm against *Staphylococcus aureus* & 11 to 21 mm against *Helicobacter pylori*. Besides, the inhibition zone diameter against *Candida albicans* ranges from 12 to 20 mm. The ADD pigment can resist microorganisms via its heteroatom, which can be adsorbed well and restrict the growth of microbes. So, the effect of ADD on the cell wall of microorganisms is due to heteroatoms. These reactions could lead to several disrubtion process as in Fig. 9:

- The ADD may interact with microbial cell membranes through the hetero atoms like N and O, increasing permeability. This disrupts the integrity of the membrane, leading to leakage of cellular contents.
- It could inhibit key enzymes involved in microbial metabolism or cell wall synthesis, preventing growth and reproduction.
- The ADD compound might generate reactive oxygen species (ROS), which can damage cellular components, including DNA, proteins, and lipids, ultimately leading to cell death.
- It may affect the synthesis or function of DNA and RNA, disrupting protein synthesis and other vital processes in microbial cells.
- The compound has chelating properties, thus it could bind essential metal ions required for microbial growth, depriving them of necessary nutrients.
- By interacting with specific metabolic pathways, the compound may hinder essential biochemical processes within the microbes.

Additionally, it is clear that the antimicrobial ability of the ADD has been improved via integration in acrylic resin rather than in the powder state. This may be attributed to the polymeric coats function as a barrier that controls the release rate of antimicrobial agents, so delaying the initial burst release of antibacterial cations and extending their duration of action^25^.

**Table 2:** The antimicrobial activity evaluation.

Strains	Coatings				
	Organic pigments (ADD)	0.1%	0.25%	0.5%	Reference
*Staphylococcus aureus*	14 ± 0.5	25 ± 0.5	26 ± 0.2	24 ± 0.5	21
*Helicobacter pylori*	11 ± 0.2	21 ± 0.1	21 ± 0.1	21 ± 0.5	22
*Candida albicans*	12 ± 0.1	19 ± 0.5	20 ± 0.2	19 ± 0.2	---

The minimum inhibitory concentration of ADD, as shown in figure (7), is 10, 11, and 11 µg/200 µL against Staphylococcus, Helicobacter pylori, and Candida albicans, respectively. While the MIC of Gentamicin is 119, 125, and 55 µg/200 µL against *Staphylococcus*,* Helicobacter pylori*, and *Candida albicans*, respectively. The findings confirm that ADD has excellent reduction ability against several microorganisms. Thus, it acts as a promising antimicrobial agent, which could be used in many applications, such as hygienic coatings, food packaging fields, and medical and pharmaceutical fields.

## Conclusions

Embedding UV-absorbing materials into acrylic resin is a way to obtain UV irradiation shielding coating radiation to the photosensitive surfaces. Herein, a synthetic ADD was integrated into waterborne acrylic resin with three proportions: 0.1%, 0.25%, and 0.5% to obtain UV irradiation-resistant and antimicrobial bifunctional coatings. SEM photos, mechanical properties, and color measurements before and after exposure to UV irradiation showed that the resistance of the films with 0.25% and 0.5% ADD to UV irradiation was very good: no significant color change or any other deterioration was observed after exposure for 500 h. The color variation (∆E) of the coating with 0.1% is around 22.5%, while (∆E) values are decreased significantly with increasing the proportions of ADD. The results confirm the integration of ADD into acrylic polymer with high ratios enhances the UV irradiation resistance, which may be attributed to the molecular structure of ADD. Besides, the antimicrobial activity of ADD powder and coatings with ADD was investigated, and the findings demonstrated that ADD powder and the coatings have excellent antimicrobial activity by performing the disc diffusion approach. The inhibition zone diameter ranged from 14 to 26 mm against *Staphylococcus aureus* & 11 to 21 mm against *Helicobacter pylori*. Also, the inhibition zone diameter against *Candida albicans* ranges from 12 to 20 mm. Benefiting from the obtained findings, the photosensitive surfaces can be protected with the prepared coatings containing ADD, which eliminates the shortcomings of the current commercial coatings with a low efficiency for absorbing UV irradiation and antimicrobial activity.

ADD-based waterborne coatings effectively address the shortcomings of traditional commercial coatings by providing superior durability, sustained antimicrobial properties, chemical resistance, environmental safety, and ease of application. This makes them a compelling choice for industries seeking reliable and sustainable coating solutions. ADD was previously used in photodiodes fabrications and is currently used to form coatings with UV irradiation-resistant having high antimicrobial activity. It can be concluded that ADD has multifunction and can be regarded as promising candidate in the future for further advanced applications.

## Data Availability

The datasets used and/or analysed during the current study available from the corresponding author on reasonable request.
